# Modulating proteasome inhibitor tolerance in multiple myeloma: an alternative strategy to reverse inevitable resistance

**DOI:** 10.1038/s41416-020-01191-y

**Published:** 2020-11-30

**Authors:** Maolin Ge, Zhi Qiao, Yan Kong, Hongyu Liang, Yan Sun, Hui Lu, Zhenshu Xu, Han Liu

**Affiliations:** 1 Shanghai Institute of Hematology, State Key Laboratory of Medical Genomics, National Research Center for Translational Medicine at Shanghai, Ruijin Hospital Affiliated to Shanghai Jiao Tong University School of Medicine, 200025 Shanghai, China; 2 State Key Laboratory of Microbial Metabolism, School of Life Sciences and Biotechnology, Shanghai Jiao Tong University, 200240 Shanghai, China; 3 SJTU-Yale Joint Center of Biostatistics and Data Science, Shanghai Jiao Tong University, 200240 Shanghai, China; 4 Fujian Institute of Hematology, Fujian Provincial Key Laboratory of Hematology, Fujian Medical University Union Hospital, 350001 Fuzhou, China

**Keywords:** Myeloma, Mechanisms of disease

## Abstract

**Background:**

Resistance to proteasome inhibitors (PIs) is a major obstacle to the successful treatment of multiple myeloma (MM). Many mechanisms have been proposed for PI resistance; however, our mechanistic understanding of how PI resistance is inevitably acquired and reversed remains incomplete.

**Methods:**

MM patients after bortezomib relapse, MM cell lines and mouse models were used to generate matched resistant and reversed cells. RNA sequencing and bioinformatics analyses were employed to assess dysregulated epigenetic regulators. In vitro and in vivo procedures were used to characterise PI-tolerant cells and therapeutic efficacy.

**Results:**

Upon PI treatment, MM cells enter a slow-cycling and reversible drug-tolerant state. This reversible phenotype is associated with epigenetic plasticity, which involves tolerance rather than persistence in patients with relapsed MM. Combination treatment with histone deacetylase inhibitors and high-dosage intermittent therapy, as opposed to sustained PI monotherapy, can be more effective in treating MM by preventing the emergence of PI-tolerant cells. The therapeutic basis is the reversal of dysregulated epigenetic regulators in MM patients.

**Conclusions:**

We propose an alternative non-mutational PI resistance mechanism that explains why PI relapse is inevitable and why patients regain sensitivity after a ‘drug holiday’. Our study also suggests strategies for epigenetic elimination of drug-tolerant cells.

## Background

The development of proteasome inhibitors (PIs) for the treatment of multiple myeloma (MM) has dramatically increased treatment responses and improved survival.^1,2^ The first-in-class PI, bortezomib, was administered intravenously (i.v.) twice-weekly as a sustained single or combination regimen.^3,4^ Bortezomib was originally used to treat relapsed MM and has subsequently been used for newly diagnosed MM. Increasing familiarity with toxicity and the optimising of dose schedules have led to the current use of PIs as key components in promoting response and eliminating resistance. Combination therapies and schedule adaptation to once-weekly use of PIs have been shown to be more tolerable and highly efficacious.^5–9^ However, the relatively rapid acquisition of drug resistance to PI treatment remains a crucial obstacle.

Innate resistance as well as acquired resistance that can arise during treatment prevents cancer therapies from achieving stable and complete responses.^10,11^ Cancer drug resistance can result from genetic mutations, but increasing evidence emphasises the contribution of non-mutational epigenetic mechanisms.^12–14^ The high frequency of epigenetic change in cancers generates a rich diversity in gene expression patterns and a heterogeneous subpopulation of cells; thus a small fraction of the cancer cell population is able to survive drug treatment as drug-tolerant cancer cells, inevitably leading to acquired drug resistance.^15–19^ Furthermore, epigenetic alterations are implicated in the loss of resistance after a ‘drug holiday’.^19–22^ However, our mechanistic understanding of PI resistance, especially the role of epigenetic regulation in the development and elimination of PI resistance, is minimal.

Initial studies have established mutations in *PSMB5* (encoding proteasome subunit β5) as the underlying cause of bortezomib resistance in vitro. However, somatic mutations of *PSMB5* are rarely detected in patients.^1,23^ Moreover, patients may display sensitivity to PIs with a relapse following initial therapy,^24–26^ implying that PI resistance is reversible. The inevitability and reversibility of PI resistance suggest a previously unrecognised drug resistance mechanism that may be intrinsic to PI treatment and regulated via epigenetic changes. However, the mechanisms underlying the reversibility of PI resistance and the effectiveness of combination and intermittent therapies have not been investigated in MM. Of particular curiosity is whether these therapies lead to the elimination of drug-resistant cells in MM.

## Methods

### Patient samples

Primary MM samples were obtained from the bone marrow of patients with diagnosed MM. Bone marrow plasma cells of patients were sorted by fluorescence-activated cell sorter with the standard method, using human CD269 (B cell maturation antigen (BCMA))-allophycocyanin (APC) antibody (Miltenyi Biotec). The purified cells were co-cultured with mesenchymal stem cells using the MesenCult-ACF Culture Kit (STEMCELL Technologies). Human primary MM samples were obtained from Ruijin Hospital (Shanghai, China) with written informed consent from each patient and research ethics board approval in accordance with the Declaration of Helsinki. The characteristics of these patients are summarised in Supplementary Table S1.

### Cell culture and generation of drug-resistant cells

Human MM cell lines MM1.S (RRID: CVCL_8792) and RPMI-8226 (RRID: CVCL_0014) were purchased from Stem Cell Bank, Chinese Academy of Sciences. Cells were authenticated using STR profiling within the past 2 years and routinely tested for mycoplasma, and all tested negative for mycoplasma. Cells were cultured in Gibco RPMI 1640 containing 10% foetal bovine serum at 37 °C with 5% CO_2_ and were maintained between a density of 5 × 10^5^ and 2 × 10^6^ cells/ml. Induced drug-tolerant cells were generated by continuously exposing parental cells to a sublethal dose of bortezomib or carfilzomib for at least 4 weeks. Cells were passaged and the inhibitors were replenished every 3 days. The half-maximal dosage effect (IC_50_) values were measured intermittently until the drug resistance was acquired. The remaining cells after the treatment were considered as ‘Tolerant’ cells and were collected for analysis. ‘Reversed’ cells were generated from bortezomib- or carfilzomib-tolerant cells by culturing without the inhibitors for a minimum of 4 weeks.

### Reagents

Bortezomib (Velcade), carfilzomib (PR-171), panobinostat (LBH589), and vorinostat (SAHA, MK0683) were obtained from Selleck Chemicals.

### Cell viability and cell proliferation assays

The CellTiter 96 MTS assay (Promega) was used to determine the cytotoxicity of the relevant drugs and cell proliferation according to the manufacturer’s instructions. For cell proliferation detection, cells were plated at the appropriate density (1 × 10^4^ cells per well of a 96-well plate), and the proliferation of the indicated cells for 5 days was detected.

### Apoptosis and cell cycle assays

Apoptosis and cell cycle were measured using the PE Annexin V Apoptosis Detection Kit and APC BrdU Flow Kit from BD Pharmingen as described by the manufacturer, respectively. Cell staining with fluorochromes were acquired using flow cytometer and data were analysed using the FlowJo software.

### Mouse studies

NOD-SCID mice were purchased from Vital River Laboratory. In all, 1 × 10^7^ MM1.S cells were subcutaneous injected into NOD-SCID mice (6–8 weeks, male or female) in the right flank. Mice were allocated randomly into different experimental groups and then administered bortezomib i.v. at 0.8 mg/kg on a twice-weekly schedule (Bortezomib^low^) or 1.3 mg/kg on a once-weekly intermittent schedule (Bortezomib^high-I^) or administered SAHA intraperitoneally at 5 mg/kg five times per week, beginning when tumours were measurable. Mice undergoing bortezomib or SAHA monotherapy also received the vehicle. No adverse events were observed. Mice were treated for 4 consecutive weeks and monitored for tumours by calliper. Mice were sacrificed by inhalation of CO_2_ when they became moribund or when their tumour diameters reached 2 cm. Animal care and sacrifice were conducted according to methods approved by the Animal Care and Use Committee, the Center for Animal Experiments of Shanghai Jiao Tong University.

### Total RNA sequencing

Total RNA was extracted from Trizol according to the manufacturer’s instructions. The mRNA-seq library was performed using the Illumina TruSeq Library Construction Kit and sequenced using BGISEQ-500RS for 100-bp paired-end sequencing. Quality control of mRNA-seq data was performed using Fatsqc, and then low-quality bases were trimmed. After quality control, clean reads were aligned to the human genome (UCSC hg19) by Tophat2.1.0 with maximum of 2 mismatches for each read. After data mapping, cufflinks were used to analyse significant differential expression genes. Gene ontology analysis was performed using the KEGG database and DAVID (http://david.abcc.ncifcrf.gov) for pathway analysis.

### Cancer hallmark analysis

We downloaded cancer hallmark data from the MSigDB database (http://software.broadinstitute.org/gsea/msigdb/). The *P* value is corrected to assess the significance of enrichment.

### Statistical analysis

Student’s *t* test was used to analyse the differences between the groups. Means were illustrated using a histogram with error bars representing ±the standard deviation (SD), and statistical relevance was evaluated using the following *P* values: *P* < 0.05 (*), *P* < 0.01 (**), or *P* < 0.001 (***).

## Results

### PIs induce reversible drug resistance in MM cells

**Fig. 1:**
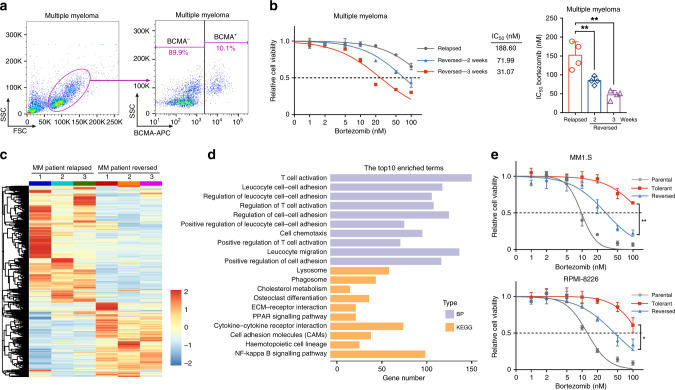
PI resistance is reversible in multiple myeloma (MM). **a** Flow cytometric detection of BCMA expression in a bone marrow sample derived from a relapsed MM patient. **b** BCMA^+^ cells were sorted and co-cultured with MSCs. Cell viability of BCMA^+^ cells was measured by MTS assay 24 h after the addition of bortezomib. The IC_50_ of different cells was quantified. One representative of four independent experiments is shown on the left and the quantitative values of the four independent experiments are shown on the right. **c** Unsupervised hierarchical clustering heatmap of differentially expressed genes (DEGs) in relapsed and reversed MM patient cells. **d** Gene ontology analysis of the biological process (BP) and KEGG pathways on data sets obtained from relapsed and reversed MM patient cells. **e** Cell viability of parental, tolerant, and reversed MM1.S and RPMI-8226 cells was measured by MTS assay 24 h after the addition of bortezomib (three independent biological replicates with three technical replicates each). *P < 0.05; **P < 0.01; two-tailed *t* test. Data are represented as mean ± SD.

To determine whether PI resistance is reversible in MM patients, we evaluated the cell sensitivity of primary MM cells. We obtained bone marrow cells from patients who relapsed after bortezomib treatment (Table S1), collected BCMA-positive MM cells,^27^ and cultured them in drug-free medium (Fig. 1a). Compared with the original relapsed cells, the cells cultured without bortezomib exhibited a remarkably enhanced sensitivity and resistance to bortezomib in these cells diminished over time (Fig. 1b), suggesting that PI resistance is reversible in MM patients. To gain insights into the molecular mechanisms underlying the reversible phenotype, we performed global mRNA expression profiling. Hierarchical clustering of the differentially expressed genes (DEGs) between the relapsed and reversed cells revealed distinct clusters of gene expression patterns (Fig. 1c). Cancer hallmark analysis showed significant enrichment of genes involved in inflammatory response pathways and cell cycle pathways (Fig. S1A). The enrichment of gene sets showed that release of inflammatory cytokines and chemokines were notably enriched (reflecting T cell activation, cell chemotaxis, cytokine–cytokine receptor interactions, and the nuclear factor-κB signalling pathway). Furthermore, there was enrichment of cell adhesion molecules (affecting regulation of cell–cell adhesion, extracellular matrix (ECM)–receptor interactions, and cell adhesion molecules; Fig. 1d and Tables S2 and S3). These biological processes and pathways correspond to soluble factor-mediated drug resistance (SFM-DR) and cell adhesion-mediated drug resistance (CAM-DR),^28^ respectively. This indicates that these resistant forms concurrently and synergistically occurred in patients with reversible relapse of MM.

To gain a more comprehensive understanding of the acquisition of this transient and reversible PI resistance, we sought to uncover the common regulatory pathways in MM patients, MM cell lines, and MM mouse models. Parental bortezomib-sensitive MM cells developed resistance to the drug when challenged with bortezomib or carfilzomib, and subsequent removal of PIs restored these cells to a relatively PI-sensitive state (Figs. 1e and S1B). These data indicate that PI treatment-refractory cells are not inherently resistant to PI but rather exist in a transient and reversible drug-resistant state. Next, we transplanted MM1.S cells into three groups of NOD-SCID mice; one group received vehicle, one group received continuous treatment with bortezomib, and the third group received bortezomib for 2 weeks and then received vehicle for 2 weeks. We further performed total RNA-seq in these MM1.S cell lines and xenograft tumours and then analysed the gene sets that changed most significantly (Fig. S2A, B). Cancer hallmark analysis revealed significant dysregulation of genes related to tumour microenvironment (TME), cell cycle pathways, and inflammatory response pathways in the PI-tolerant MM1.S cells and PI-treated MM mouse tumours (Fig. S2C, D). These dysregulated gene sets have been previously well established to be regulatory factors involved in the survival and proliferation of myeloma cells and to be associated with chemoresistance in MM patients.^28,29^ We further analysed the overlap of DEGs and gene sets in these three sequencing groups (Fig. S2E, F). On functional annotation clustering using DAVID, the 32 highest scoring clusters contained gene ontology biological process terms and KEGG pathways involving ECM organisation, ECM–receptor interaction, and cell adhesion molecules (CAMs) (Fig. S2F, G and Table S4). These data suggest that MM cells share common regulatory pathways for drug resistance in different models, including SFM-DR and CAM-DR.

### Reversible resistant MM cells are derived from slow-cycling tolerance

**Fig. 2:**
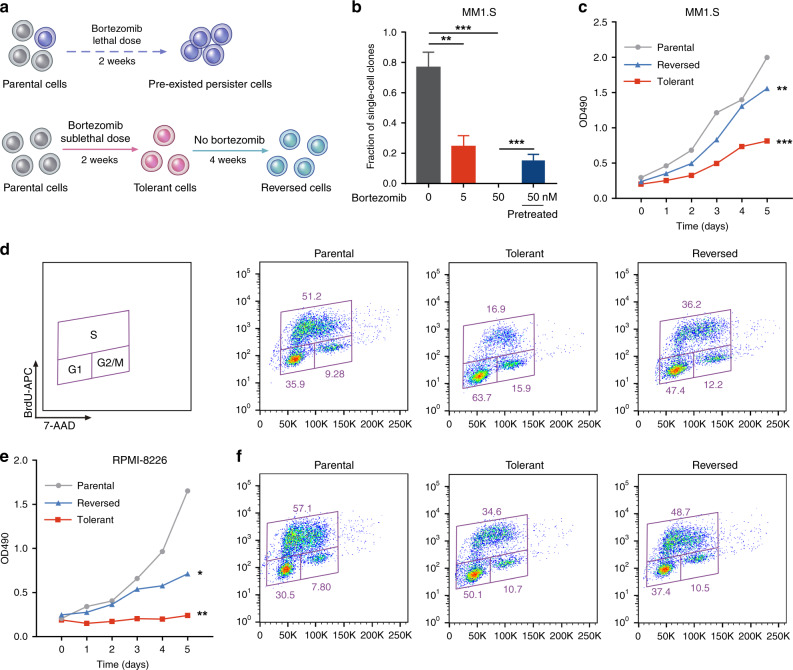
Reversible resistant MM cells are derived from slow-cycling tolerance. **a** Schematic illustrating the formation of pre-existed persister and acquired tolerant cells. **b** MM1.S single cells were sorted into individual wells and cultured at the indicated concentrations of bortezomib or pretreated with bortezomib for 4 weeks and then treated with 50 nM bortezomib. Red column, unpretreated; blue column, sublethal bortezomib pretreated. Bar plots show the fraction of single cells that form colonies (sorted single cells per condition: *n* = 288; pooled from 3 independent experiments). **c** Proliferation of the indicated MM1.S cells for 6 days (three independent biological replicates with three technical replicates each). **d** Cell cycle profiling of the indicated cells. The fraction of cells viable in G1, S, and G2/M phases of the indicated MM1.S cells is shown. **e** Proliferation of the indicated RPMI-8226 cells for 6 days (three independent biological replicates with three technical replicates each). **f** Cell cycle profiling of the indicated cells. The fraction of cells viable in G1, S, and G2/M phases of the indicated RPMI-8226 cells is shown. *P < 0.05; **P < 0.01; ***P < 0.001; two-tailed *t* test. Data are represented as mean ± SD.

An expanding body of literature emphasises the contribution of drug-tolerant or persister cancer cells in the development of inevitable drug resistance.^19^ We next sought to determine whether the reversible MM cells were derived from a fraction of pre-existent persister cells or whether, alternatively, they were induced by PI treatment (Fig. 2a). In support of the latter hypothesis, when single cells were isolated from a naive MM1.S cell population, we found that they failed to grow in the presence of a persister-tolerant dose of bortezomib (50 nM),^19^ even after extended periods of time (Fig. 2b). In contrast, when exposed to a sublethal dose of bortezomib (5 nM), a small fraction of the single cells (~20%) survived and expanded into PI-resistant populations (Fig. 2b). In addition, pretreatment of cells with the sublethal dose of bortezomib significantly increased the frequency of PI-tolerant single cells (Fig. 2b). These data suggest that the reversible MM cells are PI-tolerant cells that are acquired de novo during PI treatment and are distinct from pre-existent persister cells.

It is well recognised that under therapy stress cancer cells primarily acquire a drug-tolerant state, which is often achieved by slowing down an essential cellular process.^19^ To further characterise these PI-tolerant cells, we examined cell cycle and proliferation. Compared with parental MM cells, PI-tolerant cells exhibited a remarkably decreased proliferation and percentage of S-phase cells, and cell proliferation was significantly restored in reversed cells (Fig. 2c–f). These results suggest that PI-tolerant MM cells were slow cycling with a low S-phase fraction and were consistent with the enriched cancer hallmark analysis above (Figs. S1 and S2), indicating that MM cells enter the quiescent and tolerant state by slowing down cell cycle processes.

### PI tolerance is associated with epigenetic alterations and can be eliminated with combination and intermittent therapies

**Fig. 3:**
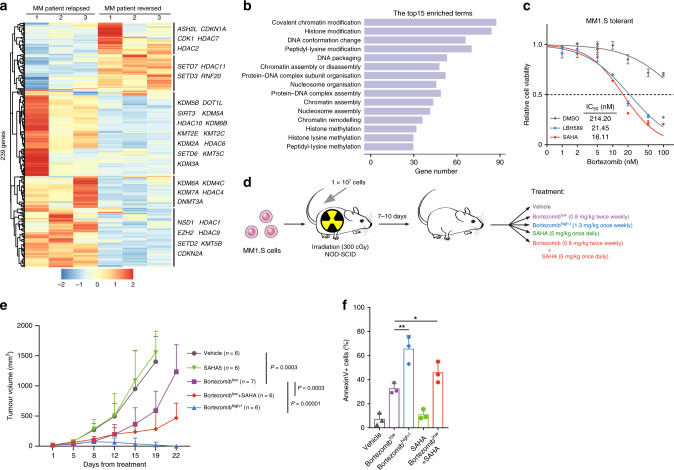
Reversible PI tolerance is mediated by epigenetic alterations and can be eliminated with combination and intermittent therapies. **a** Hierarchical clustering heatmap of the 239 differentially expressed epigenetic regulators in relapsed and reversed MM patient cells. **b** Gene ontology analysis of the BP of the differentially expressed epigenetic regulators. **c** MM1.S-tolerant cells were treated with DMSO, LBH589 (10 nM), or SAHA (2 μM) for 20 h. Cell viability was detected by MTS assay 24 h after the addition of bortezomib. The IC_50_ of different cells was quantified. **d** Treatment schedule for administration of bortezomib, SAHA, or vehicle. NOD-SCID mice were inoculated with 1 × 10^7^ MM1.S cells by subcutaneous injection in the right flank. Mice were then administered bortezomib intravenously or SAHA intraperitoneally once tumours were measurable. **e** Effect of different treatments on myeloma cell growth in vivo. Calliper measurements to estimate the tumour volume, using the following formula: 4*π*/3 × (width/2)^2^ × (length/2). **f** Tumours were obtained from sacrificed mice and were incubated with BCMA and Annexin V antibodies. Flow cytometric assays for Annexin V-positive cells in tumours are shown. *P < 0.05; **P < 0.01; two-tailed *t* test. Data are represented as mean ± SD.

Drug tolerance is a dynamic survival strategy in which individual cells transiently assume a reversible state to protect the population from eradication resulting from potentially lethal exposures. Conceivably, this reversible phenotype of drug tolerance implicates epigenetic plasticity rather than a genetic mechanism. Therefore, we analysed the differentially expressed epigenetic regulators^30^ in the expression profile of MM patients and identified a list of 239 genes (Fig. 3a). This list of genes contained many chromatin regulators, including histone methyltransferases and demethylases, histone acetyltransferases and deacetylases, as well as several other histone modification and chromatin remodelling genes (Fig. 3a). Interestingly, there were significant differences in the expression levels of several previously reported tolerance-related gene targets in cancer cells, such as *EZH2*, *KDM5*, *KDM6*, and *HDACs*.^14,17,19^ Moreover, gene sets involved in histone modification and chromatin remodelling were significantly enriched (Fig. 3b and Table S5), indicating that epigenetic alterations mediate the reversible phenotype in MM patients.

While it is widely reported that drug resistance is genetic in nature, emerging evidence in cancer biology suggests that epigenetic alterations are involved in resistance and in the loss of resistance after a ‘drug holiday’.^19^ Furthermore, in some cases, refractoriness to treatment can be reversed by epigenetic reprogramming; combination and intermittent therapies, as opposed to sustained monotherapy, appear more effective in attenuating refractoriness. The combination of PIs and histone deacetylase (HDAC) inhibitors produces synergistic cytotoxicity in preclinical MM models and in a variety of other haematological malignancy therapies.^31,32^ The HDAC inhibitors panobinostat (LBH589) and vorinostat (SAHA) are currently being tested in combination with various anticancer therapies.^31,32^ In addition, increased dosage to once-weekly use of PIs in MM patients was tested in recent clinical trials.^8^ To investigate the therapeutic basis, we further examined the effect of intermittent bortezomib-based therapy or treatment in combination with HDAC inhibitors on the drug-tolerant cells. The in vitro results demonstrated that co-treatment of MM1.S-tolerant cells with bortezomib and LBH589 or SAHA led to a strong reduction in cell viability (Fig. 3c). Next, we transplanted the MM1.S cells into NOD-SCID mice and evaluated the in vivo efficacy of the combination and intermittent therapies (Fig. 3d). Compared with the control group or SAHA single-agent treatment, engrafted mice treated with an increased dosage of bortezomib or in combination with SAHA showed notably improved responsiveness (Figs. 3e and S3A). We further analysed BCMA-positive cells by flow cytometry, and the percentage of Annexin V+ cells in different treatment conditions showed that the combination and intermittent therapies significantly decreased the number of resistant cells (Fig. 3f). Notably, intermittent bortezomib therapy reduced the number of residual cells in mice and gave the residual cells a longer time to recover, resulting in a significantly slower progression (Fig. 3e, f). These data indicate that a combination of PI with HDAC inhibitors and high-dosage intermittent therapy can prevent the emergence of PI-tolerant cells.

We next performed total RNA-seq to reveal the underlying mechanism. The gene set enrichments showed that sustained PI monotherapy was involved in inflammatory response pathways, which correspond to SFM-DR, whereas the gene sets involved in CAM-DR were enriched in both combination and intermittent therapy groups (Fig. S3B). These results indicate that intermittent bortezomib therapy or bortezomib in combination with SAHA changes the expression of downstream resistance-related genes. Moreover, the gene set enrichments and Venn diagram revealed that under the combination and intermittent therapies, a large proportion of altered epigenetic regulators in PI-tolerant cells were notably reverted (Fig. S3C, D). This indicated that these therapy strategies partly prevented or recovered epigenetic gene expression patterns. The gene set enrichments and epigenetic regulator heatmap also suggest different regulatory conditions between combination and intermittent therapies. These results strongly suggest that, with PI therapy, MM cells may have characteristic tolerant gene signatures that lead to a reversible drug resistance phenotype. Moreover, these tolerant gene signatures are no longer present in the combined or increased dosage intermittent treatment. Compared with sustained PI monotherapy, a combination therapy strategy using PIs and HDAC inhibitors or an increased dosage intermittent therapy can be more effective in treating MM by preventing the emergence of tolerant cancer cells.

## Discussion

MM is an incurable B cell malignancy because most patients eventually relapse or become refractory to current treatments. Cancer cell populations employ a dynamic survival strategy to chemotherapeutic treatments, involving a small population of drug-tolerant persister cells or cancer stem cells (CSCs) in the development of inevitable drug resistance.^15,22,33,34^ Targeting MM-CSCs is clinically relevant, and different approaches have been suggested. Currently reported drug-tolerant cells are mostly rare subpopulations that pre-existed before therapy due to transcriptional variability or epigenetic heterogeneity at the single-cell level.^17–19^ In contrast, our results confirm that PI tolerance in MM cells can be acquired de novo by bulk cancer cells through epigenetic reprogramming. Our findings suggest that drug-resistant cells can not only emerge from the treatment-mediated selection of subpopulations that present at the start of therapy but also from a general adaptation process under therapy stress. Our study also reveals the emergence and elimination of PI tolerance under treatment from an epigenetic perspective and emphasises the importance of preventing the emergence of treatment-induced drug-tolerant cells in cancer therapy. Conceivably, attenuating the acquisition of drug tolerance before the completion of the hardwiring process would be an effective way to prevent treatment failure and relapse. To this end, combination and intermittent therapy strategies, as opposed to sustained monotherapy, should be utilised to prevent cancer cells from adapting a drug-tolerant state.^35,36^

The efficacy of retreatment suggests that the reversibility of PI resistance is general in MM.^25^ However, we also need to note that our study has a limited sample quantity, especially considering the huge heterogeneity among different patients. Further studies are needed to elucidate the characteristics and discrepancies of hallmark epigenetic regulators. Moreover, despite highly encouraging preclinical data, clinical studies in patients with solid tumours failed to demonstrate any efficacy of bortezomib.^1^ Whether our proposed mechanism may extend to other cancers beyond MM remains an interesting topic requiring further investigation.

**Fig. 4:**
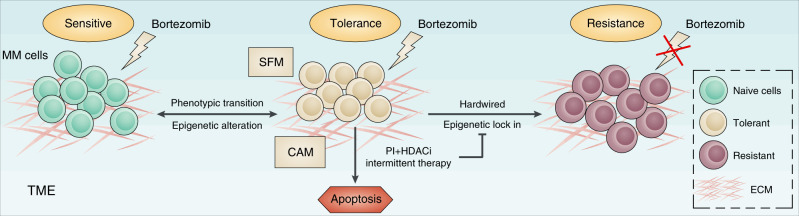
Schematic model of epigenetic regulation in the generation and elimination of PI tolerance in MM cells. PI treatment induced MM cells to enter epigenetic alteration-mediated reversible tolerance. Exposure to further chemotherapy and selection can ‘lock in’ an epigenetic state, resulting in proliferation of a hardwired drug-resistant population. However, combination with HDAC inhibitors (HDACis) and high-dosage intermittent therapy can reverse epigenetic tolerance and prevent the emergence of PI-tolerant cells. TME tumour microenvironment, ECM extracellular matrix.

Increasing familiarity with toxicity and the optimising of dose schedules has led to the current application of PIs as key components in promoting response and eliminating resistance.^5,6,9^ Furthermore, the inevitability and reversibility of PI resistance suggest a previously unrecognised drug-resistant mechanism that may be intrinsic to PI treatment and regulated via epigenetic alterations. More convenient and effective PI administration schedules have been reported to improve cancer outcomes, including MM and other haematological malignancies. Nevertheless, the reason why PI resistance is reversible and the mechanisms underlying combination and intermittent therapies have remained elusive. In this study, we propose that the appearance of drug-tolerant cells is a novel causal factor underlying reversible PI resistance in MM (Fig. 4). Our study revealed that the therapeutic basis for SFM-DR as well as for CAM-DR is the dysregulation of epigenetic regulators in MM patients during treatment. This can be eliminated by combination treatment with HDAC inhibitors and high-dosage intermittent therapy and has implications for clinical strategies to prevent the emergence of drug tolerance. Thus our study explains why combination and intermittent therapies are more effective and provides new insights that will enable more precise therapeutic strategies.

## Supplementary information

Supplementary materials

## Data Availability

RNA-seq data are available in the Gene Expression Omnibus (GEO) database under accession number GSE136725. All data described in this study are available upon request.
