# Supplementary material for: Modulating proteasome inhibitor tolerance in multiple myeloma: an alternative strategy to reverse inevitable resistance
Source: Br J Cancer. 2020 Nov 30;124(4):770–6. doi: 10.1038/s41416-020-01191-y (PMC7884794; doi:10.1038/s41416-020-01191-y)
Supplement: Supplementary file 1 — Supplementary materials [file 41416_2020_1191_MOESM1_ESM.docx]

**Supplementary Figures**

**
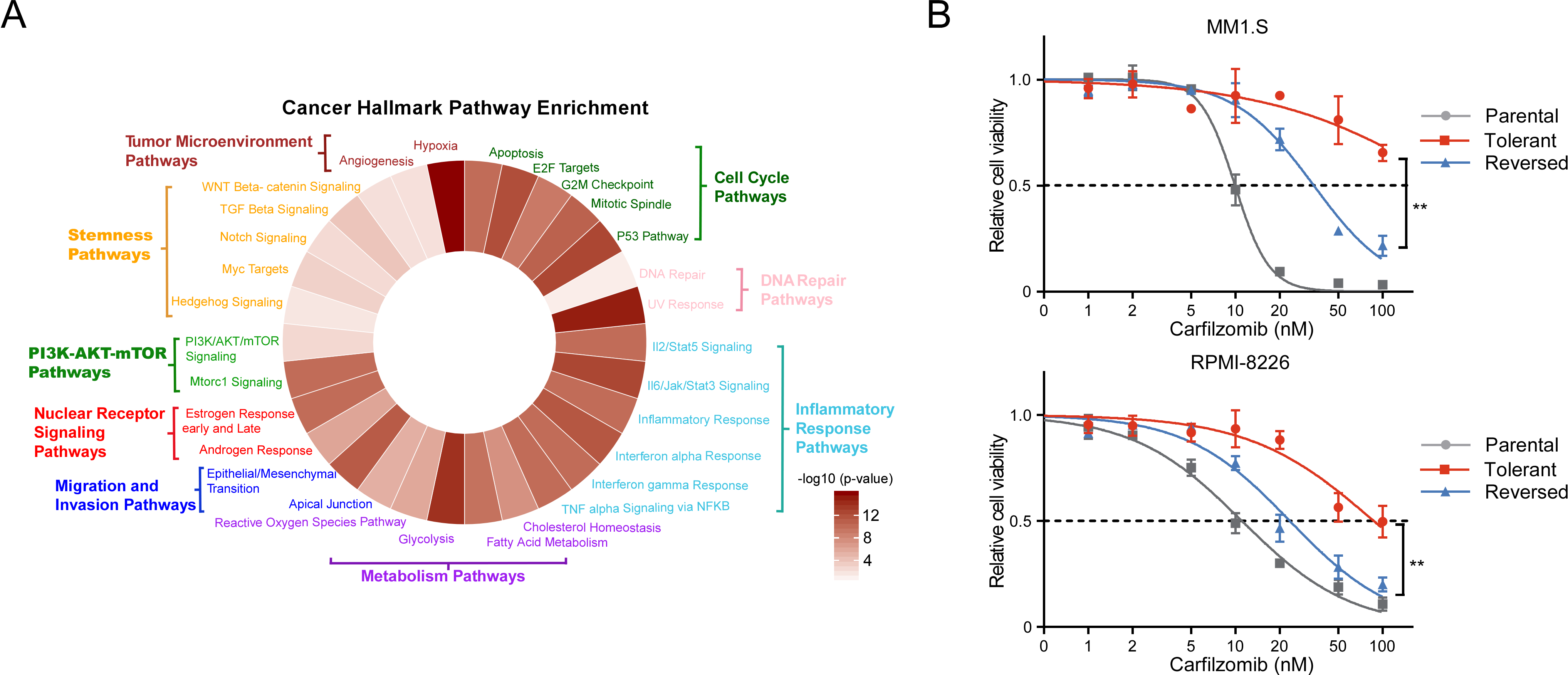
**

**Fig. S1 The reversible phenotypic transition in MM cell lines. A** Enriched cancer hallmarks generated by datasets of relapsed and reversed MM patient cells. Cancer hallmarks in the inflammatory response pathways and cell cycle pathways were significantly enriched in reversed MM cells. **B** Cell viability of parental, tolerant, and reversed MM1.S and RPMI-8226 cells was measured with MTS assay 24 hr after the addition of carfilzomib (three independent biological replicates with three technical replicates each). ** *P* < 0.01; two-tailed *t*-test. Data are represented as mean ± SD.


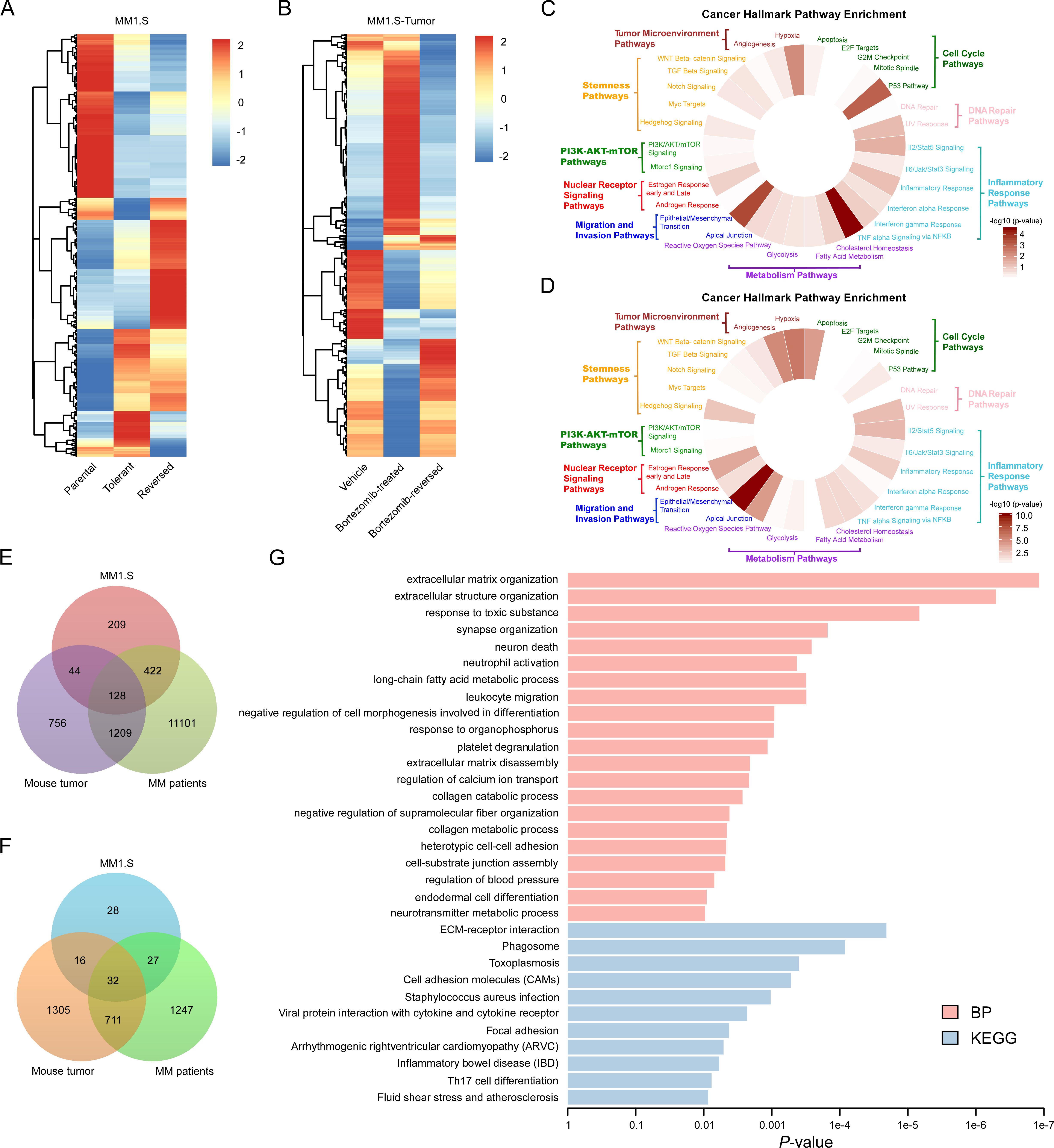


**Fig. S2 Gene set analysis of MM patients, cell lines, and mouse models.**

**A** Unsupervised hierarchical clustering heatmap of differentially expressed genes (DEGs) in the indicated MM1.S cells. **B** MM1.S cells were subcutaneous injected into NOD-SCID mice. The xenograft mice were treated with vehicle or bortezomib for 4 weeks, or withdraw bortezomib after treatment for 4 weeks. Tumor cells were collected and total RNA-Seq was performed. Heatmap of DEGs in the indicated xenograft mice is shown. **C** Enriched cancer hallmarks analysis of datasets derived from tolerant and reversed MM1.S cells. **D** Enriched cancer hallmarks analysis of datasets derived from bortezomib-treated and reversed MM1.S tumor cells. **E** Venn diagram of DEGs in MM1.S tolerant cells, bortezomib-treated MM1.S tumors, and relapsed MM patients. **F** Venn diagram of biological process (BP) and KEGG pathways in MM1.S tolerant cells, bortezomib-treated MM1.S tumors, and relapsed MM patients compared with the reversed cells. **G** The overlapped 32 BPs and KEGG pathways described in (**F**).

**
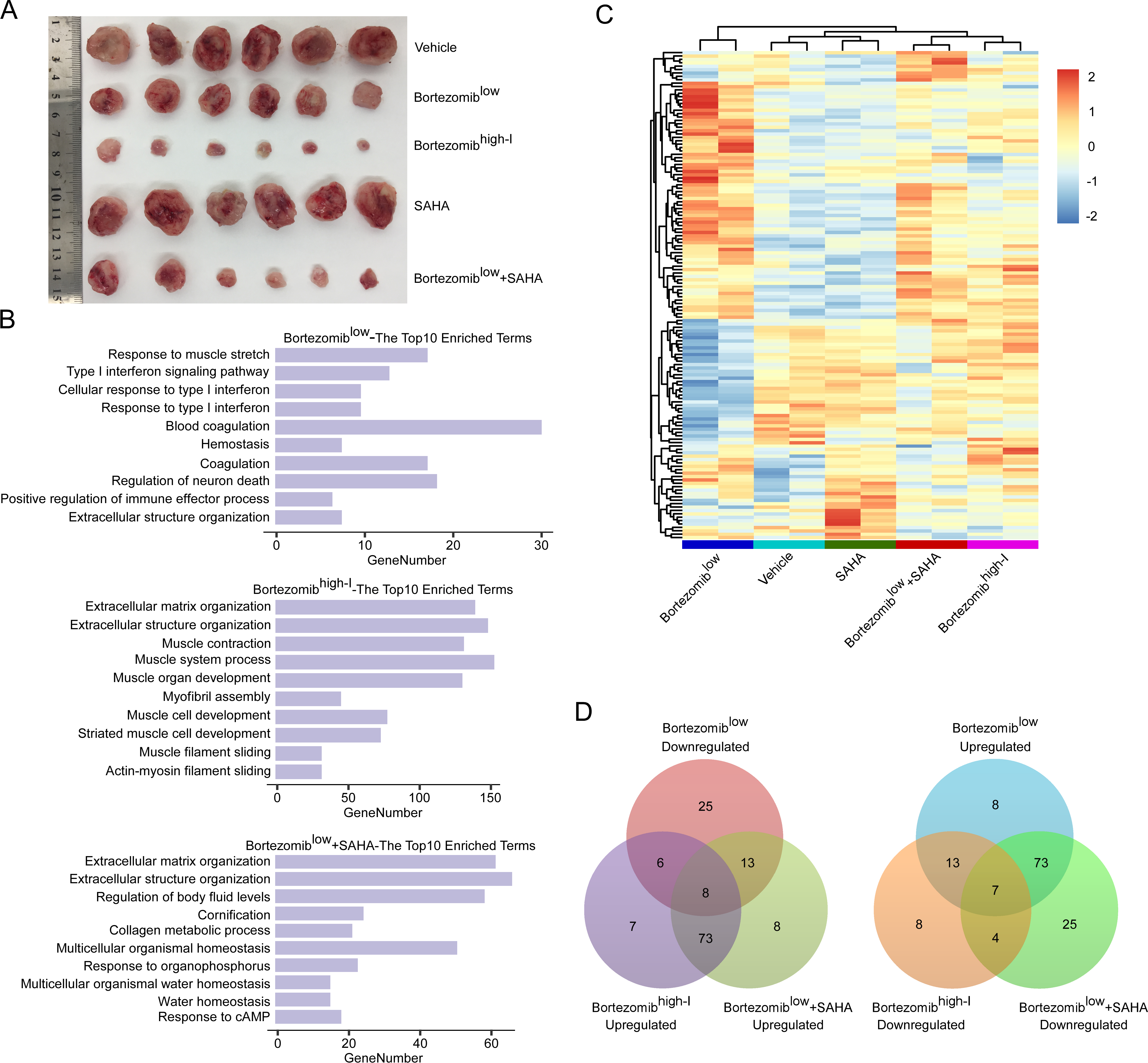
**

**Fig. S3 Therapeutic effect of combination or intermittent therapy *in vivo*.**

**A** Effect of different treatments on myeloma cell growth *in vivo*. Mice were sacrificed after treatment and their tumors were obtained. **B** Gene ontology analysis of the biological process of the differentially expressed genes in the indicated treatment groups. **C** Unsupervised hierarchical clustering heatmap of the 153 differentially expressed epigenetic regulators in xenograft mice under the indicated treatment. **D** Venn diagram of combination (Bortezomib^low^ + SAHA) and intermittent (Bortezomib^high-I^) therapies reversed downregulated (left panel) or upregulated (right panel) epigenetic regulators in MM1.S mouse model.

**Supplementary Tables**

**Table S1. Clinical characteristics of patients with multiple myeloma (MM).**

| Patient # | Sex | Pathology | Age, years | Lesion | Bone lesions^‡^ | Isotype | Heavy chain | Disease status at time of biopsy | Prior bortezomib therapy? |
| --- | --- | --- | --- | --- | --- | --- | --- | --- | --- |
| 1 | Female | MM | 61 | BM | No | λ | IgG | Relapsed | Yes |
| 2 | Male | MM | 55 | BM, EM* | Yes | κ | IgG | Relapsed | Yes |
| 3 | Male | MM | 67 | BM, EM* | Yes | κ | IgA | Relapsed | Yes |
| 4 | Female | MM | 63 | BM | No | κ | IgA | Relapsed | Yes |

* These patients exhibited extramedullary (EM) lesions. Patient #2 presented with extramedullary involvement in the lower jaw, chest skin, and liver; Patient #3 carried the infiltration lesion on the pleura.

^‡^ Bone lesions: one or more osteolytic lesions on skeletal radiography, CT, or PET-CT.

**Table S2. Top 20 enrichment of biological processes (BPs) in relapsed and reversed MM patient cells.**

| ID | Description | GeneRatio | BgRatio | p value | p.adjust | q value | Gene count |
| --- | --- | --- | --- | --- | --- | --- | --- |
| GO:0042110 | T cell activation | 149/2902 | 451/18493 | 1.91E-20 | 1.83E-16 | 1.57E-16 | 149 |
| GO:0007159 | Leukocyte cell-cell adhesion | 118/2902 | 327/18493 | 7.81E-20 | 3.73E-16 | 3.21E-16 | 118 |
| GO:1903037 | Regulation of leukocyte cell-cell adhesion | 107/2902 | 293/18493 | 1.53E-18 | 4.87E-15 | 4.19E-15 | 107 |
| GO:0050863 | Regulation of T cell activation | 109/2902 | 305/18493 | 4.61E-18 | 1.10E-14 | 9.48E-15 | 109 |
| GO:0022407 | Regulation of cell-cell adhesion | 125/2902 | 388/18493 | 2.21E-16 | 4.22E-13 | 3.62E-13 | 125 |
| GO:1903039 | Positive regulation of leukocyte cell-cell adhesion | 78/2902 | 214/18493 | 7.73E-14 | 1.23E-10 | 1.06E-10 | 78 |
| GO:0060326 | Cell chemotaxis | 97/2902 | 295/18493 | 1.29E-13 | 1.76E-10 | 1.51E-10 | 97 |
| GO:0050870 | Positive regulation of T cell activation | 74/2902 | 202/18493 | 2.49E-13 | 2.98E-10 | 2.56E-10 | 74 |
| GO:0050900 | Leukocyte migration | 136/2902 | 478/18493 | 5.79E-13 | 6.15E-10 | 5.29E-10 | 136 |
| GO:0045785 | Positive regulation of cell adhesion | 117/2902 | 397/18493 | 1.98E-12 | 1.89E-09 | 1.63E-09 | 117 |
| GO:0001819 | Positive regulation of cytokine production | 122/2902 | 422/18493 | 2.83E-12 | 2.46E-09 | 2.11E-09 | 122 |
| GO:0022409 | Positive regulation of cell-cell adhesion | 83/2902 | 251/18493 | 5.34E-12 | 4.26E-09 | 3.66E-09 | 83 |
| GO:0051249 | Regulation of lymphocyte activation | 131/2902 | 472/18493 | 1.05E-11 | 7.62E-09 | 6.55E-09 | 131 |
| GO:0070663 | Regulation of leukocyte proliferation | 74/2902 | 216/18493 | 1.12E-11 | 7.62E-09 | 6.55E-09 | 74 |
| GO:0043312 | Neutrophil degranulation | 133/2902 | 485/18493 | 1.82E-11 | 1.16E-08 | 9.98E-09 | 133 |
| GO:0002283 | Neutrophil activation involved in immune response | 133/2902 | 488/18493 | 2.90E-11 | 1.73E-08 | 1.49E-08 | 133 |
| GO:0042119 | Neutrophil activation | 135/2902 | 498/18493 | 3.08E-11 | 1.73E-08 | 1.49E-08 | 135 |
| GO:0050671 | Positive regulation of lymphocyte proliferation | 52/2902 | 132/18493 | 3.87E-11 | 2.05E-08 | 1.76E-08 | 52 |
| GO:0030595 | Leukocyte chemotaxis | 73/2902 | 217/18493 | 4.07E-11 | 2.05E-08 | 1.76E-08 | 73 |
| GO:0050866 | Negative regulation of cell activation | 65/2902 | 184/18493 | 4.39E-11 | 2.10E-08 | 1.80E-08 | 65 |

**Table S3. Top 20 enrichment of KEGG pathways in relapsed and reversed MM patient cells.**

| ID | Description | GeneRatio | BgRatio | p value | p.adjust | q value | Gene count |
| --- | --- | --- | --- | --- | --- | --- | --- |
| hsa04142 | Lysosome | 62/1402 | 123/7866 | 1.09E-16 | 3.45E-14 | 2.83E-14 | 62 |
| hsa04145 | Phagosome | 48/1402 | 152/7866 | 2.33E-05 | 3.69E-03 | 3.02E-03 | 48 |
| hsa04979 | Cholesterol metabolism | 21/1402 | 50/7866 | 5.51E-05 | 4.97E-03 | 4.07E-03 | 21 |
| hsa04380 | Osteoclast differentiation | 41/1402 | 128/7866 | 6.29E-05 | 4.97E-03 | 4.07E-03 | 41 |
| hsa04512 | ECM-receptor interaction | 27/1402 | 86/7866 | 1.33E-04 | 7.81E-03 | 6.40E-03 | 27 |
| hsa03320 | PPAR signaling pathway | 27/1402 | 76/7866 | 1.66E-04 | 7.81E-03 | 6.40E-03 | 27 |
| hsa04060 | Cytokine-cytokine receptor interaction | 77/1402 | 294/7866 | 1.73E-04 | 7.81E-03 | 6.40E-03 | 77 |
| hsa04514 | Cell adhesion molecules (CAMs) | 43/1402 | 146/7866 | 3.50E-04 | 1.38E-02 | 1.13E-02 | 43 |
| hsa04640 | Hematopoietic cell lineage | 31/1402 | 97/7866 | 4.98E-04 | 1.75E-02 | 1.43E-02 | 31 |
| hsa04064 | NF-kappa B signaling pathway | 30/1402 | 100/7866 | 6.06E-04 | 1.91E-02 | 1.57E-02 | 30 |
| hsa04061 | Viral protein interaction with cytokine and cytokine receptor | 31/1402 | 100/7866 | 8.88E-04 | 2.55E-02 | 2.09E-02 | 31 |
| hsa05144 | Malaria | 20/1402 | 49/7866 | 1.51E-03 | 3.97E-02 | 3.26E-02 | 20 |
| hsa05323 | Rheumatoid arthritis | 28/1402 | 91/7866 | 1.75E-03 | 4.25E-02 | 3.49E-02 | 28 |
| hsa00531 | Glycosaminoglycan degradation | 10/1402 | 19/7866 | 1.90E-03 | 4.29E-02 | 3.52E-02 | 10 |
| hsa04062 | Chemokine signaling pathway | 50/1402 | 190/7866 | 2.04E-03 | 4.30E-02 | 3.52E-02 | 50 |
| hsa04668 | TNF signaling pathway | 32/1402 | 110/7866 | 2.37E-03 | 4.68E-02 | 3.84E-02 | 32 |
| hsa04621 | NOD-like receptor signaling pathway | 47/1402 | 178/7866 | 2.54E-03 | 4.73E-02 | 3.87E-02 | 47 |
| hsa04659 | Th17 cell differentiation | 31/1402 | 107/7866 | 2.95E-03 | 4.99E-02 | 4.09E-02 | 31 |
| hsa04612 | Antigen processing and presentation | 24/1402 | 77/7866 | 3.00E-03 | 4.99E-02 | 4.09E-02 | 24 |
| hsa04670 | Leukocyte transendothelial migration | 32/1402 | 112/7866 | 3.24E-03 | 5.12E-02 | 4.19E-02 | 32 |

**Table S4. The overlapped 32 BPs and KEGG pathways of MM patients, MM cell lines, and MM mouse models.**

| ID | Type | Term | MM1.S | | MM patients | | Mouse models | |
| --- | --- | --- | --- | --- | --- | --- | --- | --- |
|  |  |  | p value | Count | p value | Count | p value | Count |
| GO:0030198 | BP | Extracellular matrix organization | 2.94E-06 | 23 | 7.55E-08 | 198 | 2.61E-14 | 65 |
| GO:0043062 | BP | Extracellular structure organization | 1.07E-05 | 24 | 1.67E-08 | 228 | 1.16E-13 | 70 |
| GO:0009636 | BP | Response to toxic substance | 9.05E-05 | 26 | 8.46E-06 | 272 | 7.31E-07 | 65 |
| GO:0050808 | BP | Synapse organization | 7.99E-04 | 20 | 6.58E-04 | 209 | 1.63E-10 | 64 |
| GO:0070997 | BP | Neuron death | 1.13E-03 | 18 | 1.39E-05 | 195 | 1.18E-03 | 40 |
| GO:0050900 | BP | Leukocyte migration | 2.66E-03 | 21 | 5.51E-13 | 286 | 4.76E-05 | 56 |
| GO:0001676 | BP | Long-chain fatty acid metabolic process | 3.02E-04 | 9 | 3.68E-04 | 59 | 2.07E-03 | 15 |
| GO:0042119 | BP | Neutrophil activation | 1.23E-03 | 23 | 2.62E-31 | 351 | 2.35E-03 | 52 |
| GO:0010771 | BP | Negative regulation of cell morphogenesis involved in differentiation | 2.04E-03 | 7 | 4.58E-03 | 47 | 2.76E-04 | 15 |
| GO:0046683 | BP | Response to organophosphorus | 3.05E-03 | 9 | 3.88E-03 | 74 | 8.90E-05 | 22 |
| GO:0002576 | BP | Platelet degranulation | 7.28E-04 | 10 | 9.39E-05 | 79 | 7.60E-03 | 17 |
| GO:0022617 | BP | Extracellular matrix disassembly | 2.04E-03 | 7 | 4.58E-03 | 47 | 7.47E-03 | 12 |
| GO:0051924 | BP | Regulation of calcium ion transport | 5.25E-04 | 15 | 2.37E-03 | 132 | 1.16E-02 | 27 |
| GO:0030574 | BP | Collagen catabolic process | 1.77E-04 | 6 | 4.57E-03 | 25 | 1.27E-02 | 7 |
| GO:1902904 | BP | Negative regulation of supramolecular fiber organization | 1.98E-03 | 9 | 1.19E-03 | 72 | 2.24E-02 | 15 |
| GO:0032963 | BP | Collagen metabolic process | 5.54E-04 | 9 | 2.71E-02 | 56 | 4.72E-09 | 26 |
| GO:0034113 | BP | Heterotypic cell-cell adhesion | 2.66E-03 | 6 | 9.14E-04 | 40 | 2.46E-02 | 9 |
| GO:0007044 | BP | Cell-substrate junction assembly | 1.61E-03 | 8 | 2.73E-02 | 53 | 2.16E-06 | 21 |
| GO:0008217 | BP | Regulation of blood pressure | 1.81E-03 | 11 | 3.65E-02 | 88 | 1.54E-03 | 23 |
| GO:0035987 | BP | Endodermal cell differentiation | 5.30E-04 | 6 | 1.46E-02 | 28 | 3.48E-02 | 7 |
| GO:0042133 | BP | Neurotransmitter metabolic process | 1.88E-03 | 10 | 5.16E-03 | 81 | 4.54E-02 | 16 |
| hsa04512 | KEGG | ECM-receptor interaction | 1.16E-04 | 10 | 3.44E-05 | 62 | 1.10E-04 | 18 |
| hsa04145 | KEGG | Phagosome | 7.84E-04 | 12 | 3.03E-09 | 110 | 9.32E-05 | 26 |
| hsa05145 | KEGG | Toxoplasmosis | 3.31E-03 | 9 | 7.14E-07 | 81 | 4.38E-05 | 22 |
| hsa05150 | KEGG | Staphylococcus aureus infection | 2.23E-03 | 7 | 1.42E-03 | 46 | 5.86E-04 | 14 |
| hsa05140 | KEGG | Leishmaniasis | 3.62E-03 | 7 | 7.47E-06 | 55 | 4.07E-03 | 13 |
| hsa04061 | KEGG | Viral protein interaction with cytokine and cytokine receptor | 5.38E-03 | 8 | 5.92E-03 | 62 | 4.04E-03 | 16 |
| hsa04510 | KEGG | Focal adhesion | 1.84E-02 | 11 | 7.49E-03 | 115 | 8.26E-06 | 34 |
| hsa05412 | KEGG | Arrhythmogenic right ventricular cardiomyopathy (ARVC) | 1.72E-02 | 6 | 1.26E-02 | 48 | 7.01E-04 | 15 |
| hsa05321 | KEGG | Inflammatory bowel disease (IBD) | 7.80E-03 | 6 | 3.80E-05 | 48 | 2.67E-02 | 10 |
| hsa04659 | KEGG | Th17 cell differentiation | 2.50E-02 | 7 | 8.52E-04 | 69 | 1.74E-02 | 15 |
| hsa05418 | KEGG | Fluid shear stress and atherosclerosis | 2.04E-05 | 14 | 3.83E-02 | 79 | 1.05E-02 | 19 |

**Table S5. Top 20 enrichment of BPs in relapsed and reversed MM patient cells.**

| ID | Description | Gene  Ratio | BgRatio | p value | p.adjust | q value | Gene count |
| --- | --- | --- | --- | --- | --- | --- | --- |
| GO:0016569 | Covalent chromatin modification | 86/234 | 433/17913 | 2.69E-79 | 8.60E-76 | 6.65E-76 | 86 |
| GO:0016570 | Histone modification | 83/234 | 420/17913 | 4.04E-76 | 6.46E-73 | 5.00E-73 | 83 |
| GO:0071103 | DNA conformation change | 66/234 | 279/17913 | 2.93E-65 | 3.12E-62 | 2.42E-62 | 66 |
| GO:0018205 | Peptidyl-lysine modification | 70/234 | 376/17913 | 1.45E-61 | 1.16E-58 | 8.97E-59 | 70 |
| GO:0006323 | DNA packaging | 54/234 | 194/17913 | 2.76E-57 | 1.76E-54 | 1.36E-54 | 54 |
| GO:0006333 | Chromatin assembly or disassembly | 49/234 | 178/17913 | 1.27E-51 | 6.77E-49 | 5.24E-49 | 49 |
| GO:0071824 | Protein-DNA complex subunit organization | 53/234 | 240/17913 | 2.61E-50 | 1.12E-47 | 8.67E-48 | 53 |
| GO:0034728 | Nucleosome organization | 47/234 | 165/17913 | 2.80E-50 | 1.12E-47 | 8.67E-48 | 47 |
| GO:0065004 | Protein-DNA complex assembly | 50/234 | 210/17913 | 3.46E-49 | 1.23E-46 | 9.50E-47 | 50 |
| GO:0031497 | Chromatin assembly | 45/234 | 153/17913 | 7.82E-49 | 2.50E-46 | 1.93E-46 | 45 |
| GO:0006334 | Nucleosome assembly | 43/234 | 135/17913 | 2.28E-48 | 6.62E-46 | 5.12E-46 | 43 |
| GO:0006338 | Chromatin remodeling | 38/234 | 156/17913 | 9.52E-38 | 2.53E-35 | 1.96E-35 | 38 |
| GO:0016571 | Histone methylation | 34/234 | 130/17913 | 5.65E-35 | 1.39E-32 | 1.08E-32 | 34 |
| GO:0034968 | Histone lysine methylation | 32/234 | 109/17913 | 9.31E-35 | 2.13E-32 | 1.65E-32 | 32 |
| GO:0018022 | Peptidyl-lysine methylation | 32/234 | 122/17913 | 5.39E-33 | 1.15E-30 | 8.89E-31 | 32 |
| GO:0006479 | Protein methylation | 34/234 | 169/17913 | 8.96E-31 | 1.68E-28 | 1.30E-28 | 34 |
| GO:0008213 | Protein alkylation | 34/234 | 169/17913 | 8.96E-31 | 1.68E-28 | 1.30E-28 | 34 |
| GO:0043414 | Macromolecule methylation | 39/234 | 276/17913 | 4.37E-29 | 7.76E-27 | 6.01E-27 | 39 |
| GO:0018394 | Peptidyl-lysine acetylation | 32/234 | 164/17913 | 1.50E-28 | 2.52E-26 | 1.95E-26 | 32 |
| GO:0016573 | Histone acetylation | 30/234 | 151/17913 | 4.65E-27 | 7.43E-25 | 5.75E-25 | 30 |
